# Some Practical Deductions Drawn from the Known Functions of the Liver*Read before the Chicago Medical Society, September 17, 1888.

**Published:** 1888-10

**Authors:** George W. Webster

**Affiliations:** Lecturer on Physiology in Chicago Medical College; 1922 Indiana Avenue


### The Medical Journal and Examiner.

VOLUME LVII. OCTOBER, 1888. NUMBER 4.

### ORIGINAL COMMUNICATIONS.

### SOME PRACTICAL DEDUCTIONS DRAWN FROM THE KNOWN FUNCTIONS OF THE LIVER.*

BY GEORGE W. WEBSTER, M. D.,
Lecturer on Physiology in Chicago Medical College.

It would seem like a truism to assert that a correct knowledge of physiology must be the basis of all rational therapeutics, and it is only necessary to recall the fact that prior to the delivery of the "Croonian Lectures," by the late Charles Murchison, in 1874, our knowledge of the functions of the liver was in a somewhat chaotic state, which the late Dr. Fothergill referred to as "A sort of Central Africa in the map of the organism."

While much was known before that date, and much more was surmised, clinical observations and theories had not been verified by chemistry and physiology.

But when chemistry aided physiologists to secure scientific accuracy we began to have some precise knowledge of a most important subject.

To Claude Bernard, and others of his time, we owe most of what we were taught upon this subject up to the time of his death.

If it is conceded that the most important object of physiological research is to assist in the prevention of, and recovery from, disease, the importance of the subject of this paper can not be overestimated, since in the elaboration of the food for the nutrition of the tissues, in the storing up of food in the tissues and in the liver, and of eliminating those substances deleterious to the individual, the liver is the most important glandular organ in the body.

In view of the importance of the subject, and with the hope of eliciting opinions, suggestions, and discussion which may add something to our knowledge of it, I venture to offer some thoughts in regard to it.

To treat it fully would require much time, and I shall therefore call attention to the more important functions of the liver and present some deductions drawn from them, speaking of the minor functions first.

In order to form a correct estimate of the importance of the functions of the liver, it is necessary to keep in mind the relations which the liver sustains to the digestive tract on the one hand, and to the circulatory system on the other.

We must call to mind the fact that as the portal blood stream comes from the stomach and intestinal canal, all of the products of digestion, except the fats, are poured into it—all the fluids, drugs, medicines, and everything ingested and absorbable. In this condition it comes to the liver and passes through it, and for what purpose? In the present state of our

* Read before the Chicago Medical Society, September 17, 1888.

knowledge this question can not be fully answered.

Some of the functions of the liver are quite obvious and well understood; others are not, and they are therefore matters of doubt and discussion. Progress has been made, good work has been done, much light has been thrown on an obscure subject, and the physician has been able to apply the teachings of physiology in the prevention and treatment of disease. I will now attempt to answer the question, "For what purpose does the blood pass through the liver?" The blood as it comes to the liver contains all those substances, except fats, which are necessary for the nourishment, growth, and development of the body, together with many substances of no further use in the economy, and also some which are undoubtedly injurious or poisonous, if allowed to accumulate in the blood. Under normal conditions the latter are excreted, and some of the former are changed and stored up; others are further elaborated that they may be utilized in the general constructive metabolism of the organism. When we realize how far the chemist and physicist are from grasping and interpreting all of the phenomena of inorganic matter, we cannot wonder that the intricate, difficult problems, the phenomena presented to us by living organisms, should not be well understood and our deductions which are based upon them rational. Nevertheless, we are in possession of knowledge which is of value to us if it is properly applied.

I will first state briefly the functions of the liver, the action of the bile, the glycogenic function and its elaborating action on albuminoids, and then attempt to make some practical deductions based upon a knowledge of these facts. The liver secretes bile, which is formed by the cells of the liver. It passes through the bile ducts to the gall bladder and thence into the duodenum as required in the process of digestion. During embryonic life the liver is concerned in the production of red-blood corpuscles, and in adult life it has something to do with their destruction, possibly through the action of glycogen, as glycogen possesses the power of breaking up and rapidly destroying them.

Lactic acid is formed in the liver. Deleterious substances are arrested and eliminated, and ptomaines are thought to be eliminated in this way.

The liver produces glycogen from the materials furnished it by the blood and stores it up, and again reconverts it into sugar, allowing it to pass out to meet the needs of the system.

The glycogen passes out by way of the blood vessels. We thus have the products of the activity of the liver passing out by two distinct channels, which is an exception to the general rule and our conception of a secreting organ.

The liver is the chief, and perhaps the only, organ concerned in the destructive metamorphosis of albuminoids, and the formation of urea and other nitrogenous products, which are subsequently eliminated by the kidneys, these chemical changes contributing in a marked manner to the production of heat. It also assists in the elaboration of albuminoids, fitting them for taking part in the metabolism of the tissues.

The bile is partly an excretion and partly a secretion, and after it passes into the duodenum it takes part in the process of digestion. It emulsifies fats, moistens the walls of the intestinal canal, and facilitates the passage of fats into the lacteals, since it has a certain relation to watery solutions as well as to fats. It contains a ferment which converts starch and glycogen into sugar. It excites contractions of the muscular walls of the intestines, and thus promotes absorption, and its presence increases the vital activity of the intestinal epithelium in the absorption of fats. It retards putrefactive changes in the intestinal canal, and by some it is claimed to be an antiseptic. It prevents the action of gastric juice in artificial digestion, but recent experiments seem to prove that in natural digestion in the stomach it has no such effect. Even where a fistula was formed and all the bile allowed to flow into the stomach, it did not affect the first period of digestion and did not induce vomiting as it is quite generally supposed to do.

Among the most important constituents of the bile are glycoholic and taurocholic acid, both being nitrogenous in origin and both formed in the liver by its cells from materials furnished by the blood.

That they are formed in the liver is shown by the fact that after extirpation of that organ there is no accumulation of them in the blood.

That they are nitrogenous in origin, or derived from the nitrogenous elements of the food, is shown both by their chemical composition and by the fact that they are notably increased in amount by the administration of nitrogenous food. Cholesterin is one of the principal excretory products and is kept in solution by the bile salts.

We understand by the term glycogenic function of the liver, its power to form glycogen from the materials supplied to it by the blood, and, under normal conditions, to store it up and to reconvert it into sugar to meet the requirements of the body.

Its origin is largely from the carbohydrates of the food taken, which is shown by the fact that it is increased in quantity after the ingestion of a meal rich in those substances.

It can also be formed from other classes of foods, and it is formed in minute quantities even during starvation.

When the amount of sugar in the blood exceeds five to one part in 1,000 it is eliminated by the kidneys, and the condition is known as glycosuria. When due only to excess in the blood, as after a hearty meal of carbohydrates, it is of no consequence; but when due to disease of the liver, which prevents it from converting the sugar of the food into glycogen and storing it up as such, it becomes one of the most serious and fatal of diseases.

That diabetes is a disease of the liver is shown by the fact that in an advanced stage of the disease the glycogenic function of the liver is almost abolished, and the patient becomes rapidly emaciated, because the tissues are unfed, since the sugar all slips away through the kidneys.

This is also proven, since a part of the liver has been removed from a man almost dead of diabetes, and it was found to contain almost no glycogen.

The percentage of sugar in the blood, and, therefore, the amount eliminated by the kidneys and taken away from the tissues, is increased by any condition which retards the circulation in the liver, thus allowing the ferment a longer time to act on the glycogen. This is what occurs when we puncture the lower part of the floor of the fourth ventricle, which is the hepatic vaso-motor centre, and puncture of it paralyzes the hepatic vessels. Many drugs act in the same way, notably curara, morphia, chloral, mercury, and alcohol.

Glycosuria may also be caused by chloroform narcosis, possibly by tending to cause paralysis of vaso-motor centres generally. Stimulation of the sciatic nerve may also cause it reflexly, and this may account for the fact that many people suffering from sciatica have sugar in their urine.

The increased amount of sugar in the urine may be caused by:

(1) Increased formation of sugar by the liver beyond the needs of the body.

(2) Decreased oxidation of sugar by the tissues, allowing it to accumulate in the blood.

(3) It may, as in diabetes, pass through the liver without being acted upon by the liver cells, and is, therefore, not stored up, but is eliminated by the kidneys.

The first condition may be due to a diseased condition of the liver cells or to a derangement of the circulation caused by disease of the nerves, nerve centres, or circulatory system. The third condition is evidently one of disease of the liver cells.

Glycosuria when present may be set aside in either of two ways: section of the splanchnic nerves, which renders the liver anæmic ; by the administration of glycerine, the latter substance seemingly acting on the cells of the liver, since when glycerine is administered, puncture of the floor of the fourth ventricle either does not cause diabetes, or only to a slight extent.

That the liver has an important function to perform in the oxidation and in the elaboration of albuminoids there can be no doubt.

All of the albuminoid food ingested must be converted into peptones in the alimentary canal before it can be absorbed, as albumen cannot be absorbed as such. From the time they enter the portal circulation until they emerge from the liver they again undergo further elaboration to fit them for aliment for the tissues, and to remove from them poisonous properties.

There are two kinds of proteids in the body: Those which form nitrogenous tissue or organic albumen, and those which circulate in the blood as a ready supply for the nutritive demands of the tissues, and are known as circulating albumen, or serum albumen. The latter form 3 to 4 per cent. of the blood. According to Voight, only 1 per cent. of the organic albumen is transferred in the 24 hours, while 70 per cent. of the serum albumen is transferred in the same period of time.

If all of these processes go on in a physiological manner, the albuminoids of the food take their part in the metabolism of the body and are oxidized, and the end product, urea, is finally excreted by the kidneys.

While these processes are going on in a normal manner and albuminoids are not in excess, all is well, but when they are in excess, or when they cannot be properly handled by the liver, what occurs?

Professor Albertoni has shown, according to Dr. Brunton and quoted by Dr. Fothergill, that peptones, if introduced into the general circulation, have the power to destroy the coagulability of the blood, depress and slow the heart's action, arrest the functions of the kidneys, and to cause convulsions and death.

Now, it appears from the above evidence that peptones may be poisonous, and that even though our patient have a perfect digestion as far as the stomach and the intestinal canal are concerned, if the trouble be in the liver and the peptones are not acted upon by it, we may have the injurious effects of the peptones. At the same time the tissues are unfed, the peptones are not elaborated and are drained away by the kidneys, and the individual suffers accordingly.

Again, part may pass away in this manner and the remainder undergo a retrograde metamorphosis, being changed to leucin, tyrosin, and uric acid, and thus the liability to disease of the kidneys is engendered by the elimination of waste materials, the result of this sub-oxidative process.

Again, if these waste products are not eliminated, we may have an accumulation of them in the blood, with the conditions of lithæmia, gout, nervous prostration, or even the typhoid condition.

In most forms of indigestion we are in the habit of prescribing pepsin, and various artificial digestive ferments, which are good enough when properly used; but when the fault lies, as it often does, with the liver, then we have not gone far enough.

The peptones may be formed and even absorbed, but if not further elaborated they are not only useless but may be, as has been shown, positively injurious. In this connection, Dr. Brunton says "children should not play with edge tools."

We often prescribe pepsin when we ought, instead, to stop the administration of albuminous food and do something to aid the liver.

In the metabolism of albuminoids we have the origin of many maladies, and the better we keep this in our minds the more successful will be our treatment.

We recognize the action of the liver in dealing with albuminoids, and we know now that urea is formed in the liver from the nitrogenous foods, and not in the kidneys, and that the appearance of albumen in the urine is a derangement of the liver primarily, and that the elimination of these substances may, and does, cause disease of the kidneys.

This has been well and forcibly stated by Professor Geo. S. Johnson: "Renal degeneration is a consequence of long continued elimination of waste products of faulty digestion through the kidneys."

Bright's disease is a condition of waste-laden blood, blood overcharged with nitrogenized waste, not waste representing the oxidation of the tissues, but a sub-oxidation of nitrogenous elements of food, and it matters not whether the starting point was injury to the kidneys by the attempt to excrete this mass of waste material, or whether it was due to some primary disease of the kidney cells, as in scarlatina. The treatment is the same. Begin at the source of the difficulty and stop the administration of nitrogenous food.

If "physiological rest" means anything, and is of value anywhere, it is just here; and rest for the liver and kidneys is the only way to enable them to regain lost power and to restore disordered function.

In conclusion, then, I would say, in icterus, avoid fats, as they are not absorbed on account of absence of the bile in the alimentary canal.

Where patients have inherited an inefficient liver, where they have gout, lithæmia, biliousness, an excess of bile acids, most diseases of the skin, or Bright's disease, avoid nitrogenous foods.

If we have glycosuria or diabetes, we must avoid sugars and all food which produces them.

I would like to ask the members of the society whether we can have an efficient liver as far as its glycogenic function is concerned, and yet inefficient in its elaboration of albuminoids? Do we possess drugs capable of assisting or stimulating one, without influencing the other?

1922 Indiana Avenue.

